# Molecular Targeting of Acid Ceramidase in Glioblastoma: A Review of Its Role, Potential Treatment, and Challenges

**DOI:** 10.3390/pharmaceutics10020045

**Published:** 2018-04-09

**Authors:** Ha S. Nguyen, Ahmed J. Awad, Saman Shabani, Ninh Doan

**Affiliations:** 1 Department of Neurosurgery, Medical College of Wisconsin, Milwaukee, WI 53226, USA; hsnguyen@mcw.edu (H.S.N.); aawad@mcw.edu (A.J.A.); sshabani@mcw.edu (S.S.); 2 Faculty of Neurosurgery, California Institute of Neuroscience, Thousand Oaks, CA 91360, USA; 3 Faculty of Medicine and Health Sciences, An-Najah National University, Nablus 11941, Palestine; 4 Department of Neurosurgery, University of South Alabama, Mobile, AL 36688, USA

**Keywords:** glioblastoma, acid ceramidase, acid ceramidase inhibitors, carmofur, radioresistance, radiation, sphingosine, sphingosine-1-phosphate, S1P

## Abstract

Glioblastoma is the most common, malignant primary tumor of the central nervous system. The average prognosis for life expectancy after diagnosis, with the triad of surgery, chemotherapy, and radiation therapy, is less than 1.5 years. Chemotherapy treatment is mostly limited to temozolomide. In this paper, the authors review an emerging, novel drug called acid ceramidase, which targets glioblastoma. Its role in cancer treatment in general, and more specifically, in the treatment of glioblastoma, are discussed. In addition, the authors provide insights on acid ceramidase as a potential druggable target for glioblastoma.

## 1. Introduction

Glioblastoma (GBM) is the most common, malignant primary cancer of the central nervous system in adults, with an estimated 12,120 new diagnoses in the U.S. alone in 2016. The average prognosis for life expectancy after diagnosis of GBM is less than 1.5 years; 5-year survival rate is 5% [1,2,3]. The median age of diagnosis of GBM has increased to 64 years over the last few decades, with the highest incidence within the age range 75–84 years (15.24 per 100,000 tested) [1,2,3]. Although ionizing radiation is the only well-documented cause of GBM, only a minority of patients that have been exposed to such radiation actually developed GBMs [4]. Less than 5% of patients have the germline mutation which increases the risk for developing GBMs [5,6]. For most patients, the cause of GBM remains unknown. To date, the most effective imaging method for the detection of GBM is magnetic resonance imaging (MRI), which, when combined with gadolinium contrast, provides highly detailed pictures of GBM, including its vascular supply [7].

## 2. Pertinent Clinical Features of GBM

The disease manifestation often depends on the location of the GBM. Tumors in eloquent areas tend to elicit symptoms ranging from numbness, weakness, and visual disturbance to language deficits, whereas tumors in non-eloquent areas (such as the non-dominant frontal and temporal lobes, or the corpus callosum) present with vague symptoms. The most common presenting symptoms in patients with GBM are headaches (~60%); memory loss (~40%); or cognitive, language, or motor deficits (~40%) [8]. Approximately 25% of patients with newly diagnosed GBMs experience seizures, which usually can be mitigated with anticonvulsant medications [9]. Recent data have suggested anticonvulsants may not be beneficial, and can produce significant side effects in GBM patients without seizures [10,11]. Initially, an MRI of the brain, with and without contrast, is performed for patients with symptoms that suggest brain tumors. GBMs typically exhibit a heterogeneous, ring-enhancing area of central necrosis, and infrequently can be multi-focal. Peritumoral edema, which can cause major midline shift or mass effect, could be a source of headaches and is not uncommon [12]. Treatment for peritumoral edema includes steroids, such as dexamethasone (4 mg every 6 h), which can offer significant, prompt relief from headache or deficits, generally within 48 h [13,14]. The anti-angiogenesis antibody bevacizumab may also improve peritumoral edema, but it does not improve overall survival rates in patients with newly diagnosed GBMs [15,16]. For patients who carry certain prognostic biomarker mutations such as isocitrate dehydrogenase (IDH), a new GBM may show certain characteristic features, such as a large, non-enhancing mass with pial invasion, decreased blood flow, minimal edema and necrosis, and a tendency to be present in the frontal and temporal lobes [17,18]. IDH-mutation and O6-methylguanine-methyltransferase (MGMT) promoter methylation are the two most well-studied prognostic biomarkers that show signs of improving prognoses [19,20,21,22]. To confirm the diagnosis, resected GBM tissues are formalin-fixed and paraffin-embedded prior to histopathological examinations. Under the microscope, stained GBMs exhibit palisading necrosis, marked pleomorphism, a high mitotic index, and microvascular proliferation. Additionally, they undergo immunostaining or sequencing for IDH-mutation, MGMT methylation, and other prognostic biomarkers [23,24].

## 3. Standards of Care for GBM

Treatment of newly diagnosed GBM requires a multidisciplinary approach, incorporating elements of neurosurgery, neuro-oncology, and radiation oncology. Tissue diagnosis in patients with presumed high-grade gliomas is critical. Depending on the location of the tumor, the number of lesions and/or goals of care, the treatment varies from an extensive surgical resection to a minimal biopsy. For patients younger than 70 with surgically accessible tumors, the current standard therapy includes the triad of (i.) a minimally intrusive surgical resection, (ii.) radiation therapy, and (iii.) concomitant and adjuvant temozolomide (Temodar^®^). This course of treatment is known as the “Stupp regimen” [7,25]. Exercising a higher degree of safety associated with the resection improves function and prolongs survival [26,27,28,29,30]. In addition to a surgeon’s expertise, several intraoperative techniques are used to maximize safety during the surgical resection. Awake craniotomy, with intraoperative cortical stimulation and neuropsychological assessment, improves maximal resection and reduces postoperative neurologic and language dysfunction [31]. Intraoperative imaging, such as CT (computed tomography) or MRI, is another strategy for achieving a maximally-safe resection, as it provides prompt identification of residual tumors. In a prospective randomized trial, the intraoperative MRI group had significantly higher rates of complete tumor resection, without increased rates of postoperative neurological deficits, compared with the group with which no intraoperative imaging was used [32]. Lastly, fluorescence-guided surgery is a new method that helps visualize malignant tissue during surgery. 5-aminolevulinic acid (5-ALA) is a photosensitizer agent used guide glioma resection; its use was recently approved by the U.S. Food and Drug Administration. With the use of 5-ALA, several studies have demonstrated higher rates of maximal resection, with possible extended progression-free survivals [33,34,35].

Several analyses have shown more survival benefit with a radiation dose of 60 Gy, compared to 45 Gy. No improvement was noted at 70 Gy [36,37,38]. In addition, stereotactic radiation offers no additional benefits when added to the standard regimen of 60 Gy [39]. Currently, most patients younger than 70 receive 60 Gy in 30 fractions over a 6-week period. For patients older than 70 years, a study showed that 50.4 Gy was beneficial and improved median survival from 16.9 to 29.1 weeks [40]. Compared with a standard 6-week course of radiation, a shorter 3-week course of radiation may provide a better outcome to the elderly as well [41,42].

Stupp and colleagues recently reported the results of a randomized clinical trial using tumor-treating fields (TTFs), which supply alternating electric currents through an array of electrodes applied to the shaved scalp for >18 h a day, to treat patients with newly diagnosed GBM [43,44]. TTFs are believed to disrupt cell division by preventing proper formation of mitotic spindles, causing rapid disintegration of dividing cells [45]. TTFs improved median overall survival to 24.5 months (from 19.8 months) [43,44].

Molecular characteristics of GBM have recently gained much attention due to their role in prognosis and treatment. Several mutations have demonstrated their role as independent prognostic factors. IDH mutations (IDH-1 and IDH-2), MGMT status, and telomerase reverse transcriptase gene mutations should be tested in young patients with GBM, due to their prognostic relevance [7].

In our practice, we utilize awake craniotomy and intraoperative MRI to maximize safe surgical resection of GBMs. To help with prognostication, we routinely test most, if not all, GBM tissues for mutations or changes in the aforementioned prognostic biomarkers. We also offer TTFs to patients who are willing to wear the device for at least 18 h per day.

## 4. ASAH1 and Its Role in Cancer

**Figure 1:**
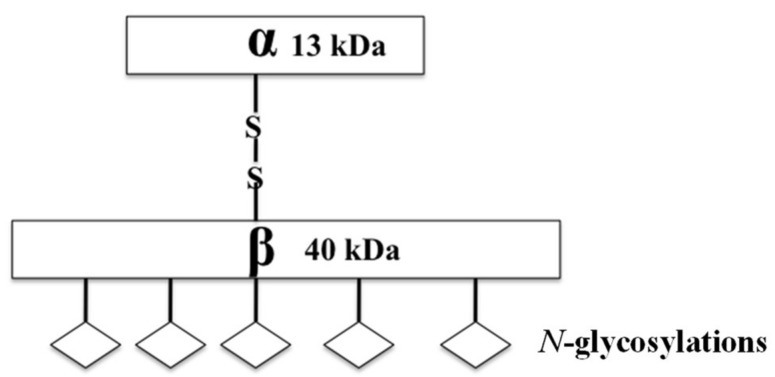
A cartoon diagram shows the mature structure of ASAH1, including the disulfide linked α- and β-subunits and their molecular weights.

**Figure 2:**
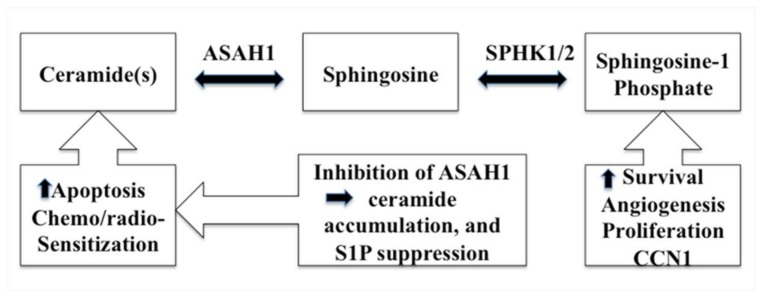
A schematic diagram describes the ceramide metabolism pathway and the resulting effects of inhibiting ASAH1.

Acid ceramidase (ASAH1) is a lysosomal cysteine amidase that catalyzes the transformation of ceramide into sphingosine and free fatty acid (Figure 1) [46,47,48]. Subsequently, sphingosine kinase 1 (SPHK1) or 2 (SPHK2) phosphorylate sphingosine forms the tumor promoter, sphingosine-1-phosphate (S1P) (Figure 2) [46]. ASAH1, initially discovered in rat brain homogenates and further characterized and purified from human urine in 1995, has a molecular weight of 53–55 kDa, depending on the extent of glycosylation. It is composed of α- and β-subunits, 13 kDa and 40 kDa in size respectively, that are linked by disulfide bonds (Figure 1) [49,50,51]. *N*-Glycosylations occur exclusively on the β-subunit, while the α-subunit is not glycosylated (Figure 1) [51,52]. Initially synthesized as a pro-enzyme ~55 kDa, ASAH1 then undergoes autoproteolytic activation, a step that requires essential nucleophile cysteine residue 143 to cleave ASAH1 into the α- and β-subunits within lysosomes [53,54]. Fitting with its cellular localization, its enzymatic activity has an optimal pH range between 4.0 and 5.0 [55]. Although ASAH1 is a lysosomal protein, a portion of ASAH1 is also secreted, as seen in fibroblasts [51,52,55].

Increasingly cogent papers have linked the sphingolipid pathway to cell proliferation and the development of resistance to anticancer therapies. Studies have revealed that S1P promotes GBM invasiveness via the upregulation of the urokinase plasminogen activator, its receptor, and pro-invasive molecule CCN1 (cysteine-rich angiogenic protein 61) (Figure 2) [56,57]. Conversely, a high level of ceramide induces apoptosis in cells that have undergone radio- and chemotherapy via the release of cytochrome c, leading to the activation of caspase-9 and caspase-3 (Figure 2) [46,47,48,58,59]. Ceramides, carrying fatty acid side chains ranging from 14 to 26 carbons, are generated via the action of ceramide synthases (CerS), which comprise a family of 6 enzymes [60,61,62]. Each specific ceramide species can elicit unique cellular responses [63]. Apoptosis has been attributed to CerS6, because the inactivation of CerS6 expression decreases both the level of C_16_-ceramide and the cellular susceptibility of cells to the death receptor ligand TRAIL. Conversely, an overexpression of CerS6 increases susceptibility to TRAIL [60]. Interestingly, ASAH1 transcription is augmented when the expression of CerS6 is repressed [64]. Because its products are involved in the regulation of cell proliferation, multiple cancers have been linked to ASAH1, such as melanoma, acute myeloid leukemia (AML), and colon and prostate cancers [65,66,67]. In a genomic study, ASAH1 was found to be a useful biomarker for cancer detection and identification in cases of melanoma [68]. Recently, Lai et al. reported that ablation of ASAH1 inhibits cancer-initiating cell formation, as well as self-renewal of invasive melanoma [69]. ASAH1 activity is also deregulated in AML. Consequently, ASAH1 has been put forward as an emerging drug target in AML [70]. Investigators reported that, in prostate cancer, over-expression of ASAH1 leads to larger tumor volumes that are more resistant to chemotherapy. Also, when ASAH1 is suppressed, cells become more sensitive to chemotherapy, and the treatment with B13 (1*R*,2*R*)-2-(tetradecanoylamino)-1-(4′-nitrophenyl-1,3-propandiol), an ASAH1 inhibitor, sensitizes these cells to radiation (Table 1) [66,71]. ASAH1 is overexpressed in 67% of human prostate tumors vs 70% of head and neck cancers when compared with normal controls [72].

In a bid to develop new drugs, researchers have investigated the effect of inactivating ASAH1. Inhibiting ASAH1 activity resulted in the accumulation of intracellular ceramide up to cytotoxic levels, that induced a significant amount of apoptosis in SW403 human adenocarcinoma cells when exposed to the ASAH1 inhibitor B13 (Figure 2) [73]. Similarly, acid ceramidase inhibitor ceranib-2 induces apoptosis via the activation of stress-activated protein kinase/c-Jun N-terminal kinase, p38 mitogen-activated protein kinase apoptotic pathways, and inhibition of the Akt pathway in breast cancer cell lines MCF-7 and MDA MB-231 [74]. Ceranib-2 has also been found to have a synergistic effect with carboplatin in targeting non-small cell lung cancer cells [75]. Carmofur, a well-studied ASAH1 inhibitor, has been shown to have antiproliferation and antimetastatic properties against cervical cancers, and the Wnt/β-catenin signaling pathway seems to play a role [76,77]. More clinically relevant is that the postoperative adjuvant use of carmofur in patients with early breast cancer appears to be beneficial [78]. On the other hand, activating ASAH1 in hepatoma cells resulted in higher levels of intracellular S1P, lower levels of ceramide, and increased cell survival compared with controls, as found by Hara et al. (Figure 2) [79]. Furthermore, treating trophoblasts with the ASAH1 inhibitor abrogated the antiapoptotic effect of the epidermal growth factor, resulting in ceramide-induced apoptosis [66]. As a potent driver of cell growth and invasion, ASAH1 enables cells to become more resistant to tumor-necrosis-factor-induced apoptosis, and to migrate more rapidly [66].

## 5. ASAH1 in GBM

In an effort to study the invasion mechanism of GBM, Annabi et al. demonstrated that a population of CD133^+^ (a stem cell marker) cells isolated from the U87MG parental cell line increased migratory response to S1P compared with parental U87MG cells, possibly through a combined pathway of S1P /LPA (sphingosine-1-phosphate/lysophosphatidic acid) cell surface receptors signaling and by membrane-type-1 matrix metalloproteinase [80]. Following this, it was shown that in histologically confirmed glioma specimens, there was a shift from ceramide to S1P that increases with glioma cancer grade, and that glioblastoma tissues carry 9-fold higher S1P and 5-fold lower ceramide concentrations compared to normal gray matter [81]. Our recent data suggest the higher level of S1P may be due to an elevated level of ASAH1, which converts ceramide to S1P (Figure 2) [82]. Consistent with this, we demonstrated the negative effect of ASAH1 on GBM survival via Western blotting and immunohistochemistry studies [82]. In addition, the regulation of ASAH1 may have been altered in GBM, allowing it to be secreted possibly into the interstitial tissues, and therefore, may enable GBM ASAH1-secreting cells to transfer their malignant potential to nearby cells [82]. Increased secretion of ASAH1 was also found in prostate cancer, but not in nonmalignant cells, affording a certain capacity to distinguish between prostate cancer and nonmalignant cells [82]. We propose that ASAH1 may decrease overall GBM survival by enhancing survival of CD133+ cells, therefore producing resistance to common anticancer therapies [82]. As a result of developing resistance to therapy, GBM almost always recurs. ASAH1 may play a role in developing resistance to radiation. Prostate cancer upregulates ASAH1 following radiation, which was described as a mechanism enabling the cancer to survive radiation [83]. The ASAH1 level was similarly elevated in irradiated U87 cells or tissues of GBM patients who received radiation, compared with controls (Table 2) [84]. Interestingly, secretion of ASAH1 was also increased following radiation (Table 2) [84]. This elevation of ASAH1 appears to confer radioresistance to GBM cells by decreasing the level of proapoptotic ceramide molecules, and increasing the level of prosurvival S1P molecules, and may contribute to recurrence (Table 2) [84]. Similarly, ASAH1 is expressed in the pediatric GBM cell lines and is upregulated following radiation [84,85].

## 6. Molecular Targeting of ASAH1 and Challenges

The increased apoptosis in U87 cells when exposed to an ASAH1 inhibitor was demonstrated in a study by Hara et al., which also revealed an elevation in ceramide as the likely mechanism [79]. Etoposide, a chemotherapy drug previously used to treat brain tumors, promotes glial apoptosis in the C_6_ glioma cell line via the activation of caspase-9 and caspase-3 mediated by the ceramide pathway, which stimulates the excretion of cytochrome c [58]. ASAH1 inhibitors can also function as radiosensitizers, as suggested by a study that illustrated the suppressed growth of prostate cancer xenografts compared with conventional radiation therapy when mice were given ASAH1 inhibitor B13 (Table 2) [71]. In particular, the treatment of the adult GBM U87 with an ASAH1 inhibitor sensitizes these cells to radiation [79]. To extend these therapeutics studies further, ASAH1 inhibitors such as carmofur, *N*-oleoylethanolamine, and ARN14988 (C_16_H_24_ClN_3_O_5_) were tested against U87 and multiple glioma stem-like cells (Table 1). These inhibitors were highly effective against all of these cell lines, with the increasing ceramide level and decreasing S1P level as the likely mechanism mediating apoptosis (Figure 2 & Table 1) [82]. A significant increase in apoptosis was also seen when testing these ASAH1 inhibitors against pediatric GBM cell lines, suggesting that ASAH1 is a novel drug target (Table 1) [85]. Among these studied ASAH1 inhibitors, carmofur is the only drug that has been used clinically: it was first used in Japan in 1981 for the treatment of colorectal cancers [86,87,88]. Although carmofur is capable of penetrating the blood-brain barrier, the extent to which it is able to penetrate brain tumors remains unknown [77]. Carmofur is highly insoluble in aqueous solution, and an intravenous formula is unavailable. Due to this limitation, further optimization and characterization of carmofur to improve its solubility and brain tumor penetration is necessary. To this end, Realini et al. and Ortega et al. have performed a significant amount of work to develop more effective derivatives of carmofur, with promising results (although their ability to cross the blood-brain barrier has not been demonstrated) [77,89,90]. To improve solubility and drug delivery, carmofur was embedded in nanogel. The nanogel, when injected into mice, demonstrates improved suppression of tumor growth compared with carmofur solution [91]. Coating the carmofur-encapsulated nanogel with polysorbate 80 results in an improved penetration of the blood-brain barrier [92]. An ASAH1 inhibitor that specifically targets the lysosome has also been developed. For more specific treatment of lysosomal ASAH1, B13 is conjugated to lysosomotropic *N*,*N*-dimethyl glycine (DMG), to form prodrug LCL521 (1,3-*di*-DMG-B13), with the enhanced capability of inhibiting cell growth through specific targeting of lysosomal ASAH1 (Table 1) [93,94]. Perhaps a structure-based drug design technique can be employed to facilitate development of a highly specific inhibitor against ASAH1. This process would require either the crystal or nuclear magnetic resonance structure of ASAH1 to be identified to accurately define the active site, in order to design the most appropriate inhibitor. Alternatively, the use of antibodies to target secreted ASAH1 and S1P has also been shown to effectively slow the rate of cell proliferation in U87 [84]. This finding opens a new means of targeting glioma through immunotherapy, in order to limit cell proliferation and invasion. Potentially, immunizing patients against ASAH1 would specifically target extracellular ASAH1 while leaving intracellular ASAH1 intact, and may limit GBM proliferation and invasion. In fact, the benefit of developing an auto-ASAH1 antibody has already been documented in melanoma patients. It was found that melanoma patients who had an auto anti-ASAH1 antibody were protected from lymph node metastasis. Upregulation of an auto anti-ASAH1 antibody may mitigate melanoma metastasis; the loss of this antibody may result in melanoma progression [95]. Further investigation is needed to determine if a similar protective benefit against tumor progression can be seen in GBM patients that developed auto anti-ASAH1 antibodies through active immunization.

## 7. Conclusions

The prognosis of GBM remains poor despite all current treatments being employed. To improve GBM outcomes, more new drugs with novel targets in GBM are urgently needed. ASAH1 has been shown to be an important drug target in regulating the growth of GBM cells and glioma like-stem cells. Inhibiting ASAH1 with carmofur in vitro has shown promising results. However, carmofur is difficult to handle due to its solubility issues, and this has hampered the study of the drug in vivo. Development of a more water-soluble, potent derivative of carmofur that can penetrate the blood-brain barrier is essential to study its efficacy in vivo. Alternatively, employing immunotherapy or a tumor vaccine against secreted ASAH1 may also be an important strategy to be considered in future studies.

## Figures and Tables

**Table 1:** Cell lines that were tested with various ASAH1 inhibitors are shown, including their chemical compositions.

Cell Lines Tested	Carmofur (C_11_H_16_FN_3_O_3_)	*N*-oleoylethanolamine (C_20_H_39_NO_2_)	B13 (C_23_H_38_N_2_O_5_)	ARN14988 (C_16_H_24_ClN_3_O_5_)	LCL521 (1,3-*N*,*N*-dimethyl glycine-B13)
**SJGBM2**	x	x			
**SJGBM2-10gy**	x				
**CHLA259**	x	x			
**CHLA200**	x	x			
**CHLA266**	x	x			
**U87**	x	x		x	
**U87-10gy**	x	x			
**GSC 22**	x	x		x	
**GSC 33**	x	x		x	
**GSC 44**	x	x			
**SW403**	x				
**LNCaP**	x		x		
**HEK 293**	x				
**MCF7**			x		x
**PC3**			x		
**A375**				x	
**G361**				x	
**M14**				x	
**MeWo**				x	
**MNT-1**				x	
**SkMEL28**				x	

**Table 2:** Cellular changes in GBM following radiations are shown.

Cellular Changes Following Radiation
Increased secretion of ASAH1
Upregulation of intracellular ASAH1
Decreased intracelular level of ceramides
Increased intracellullar level of S1P

Acid ceramidase: ASAH1, Sphingosine-1-phosphate: S1P.

## Abbreviations

5-ALA	5-aminolevulinic acid
ASAH1	acid ceramidase
AML	acute myeloid leukemia
CerS	ceramide synthases
DMG	*N*-dimethyl glycine
GBM	glioblastoma
IDH	isocitrate dehydrogenase
MGMT	O6-methylguanine-methyltransferase
MRI	magnetic resonance imaging
S1P	sphingosine-1-phosphate
SPHK1	sphingosine kinase 1
SPHK2	sphingosine kinase 2
TTF	tumor-treating field
